# High-viscosity sample-injection device for serial femtosecond crystallography at atmospheric pressure

**DOI:** 10.1107/S1600576719012846

**Published:** 2019-10-17

**Authors:** Yoshiaki Shimazu, Kensuke Tono, Tomoyuki Tanaka, Yasuaki Yamanaka, Takanori Nakane, Chihiro Mori, Kanako Terakado Kimura, Takaaki Fujiwara, Michihiro Sugahara, Rie Tanaka, R. Bruce Doak, Tatsuro Shimamura, So Iwata, Eriko Nango, Makina Yabashi

**Affiliations:** a RIKEN SPring-8 Center, 1-1-1 Kouto, Sayo-cho, Sayo-gun, Hyogo 679-5148, Japan; b Japan Synchrotron Radiation Research Institute, 1-1-1 Kouto, Sayo-cho, Sayo-gun, Hyogo 679-5198, Japan; c Department of Cell Biology, Graduate School of Medicine, Kyoto University, Yoshidakonoe-cho, Sakyo-ku, Kyoto 606-8501, Japan; d Department of Biological Sciences, Graduate School of Science, The University of Tokyo, 2-11-16 Yayoi, Bunkyo, Tokyo 113-0032, Japan; e Max Planck Institute for Medical Research, Jahnstrasse 29, 69120 Heidelberg, Germany

**Keywords:** serial femtosecond crystallography, X-ray free-electron lasers, room-temperature crystallography, microcrystal injection

## Abstract

A sample-injection device has been developed at SPring-8 Angstrom Compact Free-Electron Laser (SACLA) for serial femtosecond crystallography (SFX) at atmospheric pressure. Microcrystals embedded in a highly viscous carrier are stably delivered from a capillary nozzle with the aid of a coaxial gas flow and a suction device. The cartridge-type sample reservoir is easily replaceable and facilitates sample reloading or exchange. The reservoir is positioned in a cooling jacket with a temperature-regulated water flow, which is useful to prevent drastic changes in the sample temperature during data collection. This work demonstrates that the injector successfully worked in SFX of the human A_2A_ adenosine receptor complexed with an antagonist, ZM241385, in lipidic cubic phase and for hen egg-white lysozyme microcrystals in a grease carrier. The injection device has also been applied to many kinds of proteins, not only for static structural analyses but also for dynamics studies using pump–probe techniques.

## Abstract 2

A high-viscosity cartridge-type injector for serial femtosecond crystallography has been developed at SPring-8 Angstrom Compact Free-Electron Laser.

## 1. Introduction   

Serial femtosecond crystallography (SFX) is a recently developed technique for determining protein crystal structures using X-ray free-electron lasers (XFELs) (Chapman *et al.*, 2011 ▸; Boutet *et al.*, 2012 ▸). Whereas conventional X-ray diffraction data collection using synchrotron sources requires measurements at cryogenic temperatures to prevent radiation damage, SFX allows data collection at room temperature because intense femtosecond XFEL pulses afford diffraction patterns from protein microcrystals before the onset of radiation damage (Neutze *et al.*, 2000 ▸). Given that microcrystals are destroyed by a single XFEL pulse, replenishment of intact crystals is imperative for data collection in SFX. In general, crystals are suspended and injected using a carrier medium such as buffer solution as a thin sample stream of several micrometres to several tens of micrometres. The crystal concentration governs the probability of the XFEL pulse hitting crystals in the carrier medium. Given the measurement efficiency, a crystal density of 10^7^–10^8^ crystals ml^−1^ is necessary to obtain a complete SFX data set, which comprises still diffraction patterns from randomly oriented crystals. However, a high crystal concentration causes clogging of the injector nozzle. In addition, this approach consumes large quantities of the sample because most of the crystals do not intersect with the XFEL pulses. The stable delivery of microcrystals into an interaction point of the XFEL that consumes a reasonably small amount of protein remains a technical challenge. In order to overcome the issue of sample delivery, several sample-injection methods have been developed. A gas dynamic virtual nozzle (GDVN) utilizes a helium sheath gas flow for focusing the liquid jet consisting of crystal suspensions in buffer solutions to reduce sample consumption (DePonte *et al.*, 2008 ▸). In another liquid-jet injection method, a microfluidic electrokinetic sample holder (Sierra *et al.*, 2012 ▸) is based on a principle utilized in electrospinning. Alternatively, a highly viscous carrier medium, such as lipidic cubic phase (LCP), has been used for extruding samples with a slow flow rate (Weierstall *et al.*, 2014 ▸).

Injection methods that use highly viscous carriers have the great advantage of being able to reduce sample consumption for SFX because they have lower flow rates than liquid-jet methods. Typically, highly viscous samples with a concentration of 10^7^–10^8^ crystals ml^−1^ are delivered at a flow rate of 0.25–0.5 µl min^−1^, whereas a GDVN often requires a sample flow rate of the order of 10 µl min^−1^ to create a stable jet of crystal suspensions at concentrations of 10^8^–10^9^ crystals ml^−1^. For example, an SFX structure of the human serotonin 5-HT_2B_ receptor was determined using only 300 µg of protein crystallized in LCP (Liu *et al.*, 2012 ▸). The LCP crystallization method utilizing lipids such as monoolein is suitable for producing highly ordered tiny crystals of membrane proteins (Landau & Rosenbusch, 1996 ▸; Caffrey & Cherezov, 2009 ▸). Crystals in LCP can be loaded into a sample injector as produced since gel-like LCP material works as a highly viscous carrier. For extruding samples produced by vapor diffusion or batch crystallization, grease (Sugahara *et al.*, 2015 ▸) is a versatile hydro­phobic medium. For instance, the structure of lysozyme was determined by a grease matrix method from only 100 µg of protein (Sugahara *et al.*, 2015 ▸). Petroleum jelly (Botha *et al.*, 2015 ▸) is also useful as a hydro­phobic carrier medium for sample delivery in SFX. To reduce the background noise and/or extend the applicability of the highly viscous carrier methods, hydro­philic media such as agarose (Conrad *et al.*, 2015 ▸), hyaluronic acid (Sugahara *et al.*, 2016 ▸), cellulose derivatives (Sugahara *et al.*, 2017 ▸; Kovácsová *et al.*, 2017 ▸), Pluronic F-127 (Kovácsová *et al.*, 2017 ▸) and high-molecular-weight poly(ethyl­ene oxide) (Martin-Garcia *et al.*, 2017 ▸) have also been employed in SFX experiments.

For the injection of highly viscous samples, an LCP injector was developed by Weierstall *et al.* (2014 ▸). This injector is composed of a hydraulic cylinder driven by a high-performance liquid chromatography (HPLC) pump, a sample reservoir and a nozzle section. Coaxial gas flow along the inner capillary in the nozzle section keeps the LCP stream on-axis. In the initial applications of the LCP injector at the Linac Coherent Light Source, microcrystals in LCP were extruded in a vacuum chamber at ∼10^−3^ Torr, in which background scattering from ambient gas was fully suppressed. However, the evaporative cooling of the sample under vacuum led to a transition of LCP into a lamellar crystalline phase, affording strong diffraction rings (Weierstall *et al.*, 2014 ▸). This phase transition has typically been prevented by the addition of 7.9 mono­acyl­glycerol [MAG: 1-(7*Z*-hexadecenoyl)-*rac*-glycerol] or 9.7 MAG [1-(9*Z*-hexadecenoyl)-*rac*-glycerol] to the sample (Liu *et al.*, 2014 ▸). However, these lipids are known as precious lipids because they cost five to nine times more than monoolein. Thus, the suppression of a drastic change in the sample temperature during the injection process is desirable. In addition, a vacuum environment offers less flexibility for installing devices or mounting a sample injector. Recently, an atmospheric pressure environment (helium or air) was introduced for SFX experiments to increase flexibility (Tono *et al.*, 2015 ▸; Sierra *et al.*, 2019 ▸).

Here we describe the development of a high-viscosity sample-injection device for SFX experiments under atmospheric pressure. The sample-injection device consists of a high-viscosity cartridge-type (HVC) injector and a suction device. To demonstrate the performance of the sample-injection device, SFX experiments were carried out using LCP-grown microcrystals of the human A_2A_ adenosine receptor (A_2A_AR) complexed with a non-xanthine antagonist, ZM241385, and on hen egg-white lysozyme microcrystals in a grease carrier matrix at SPring-8 Angstrom Compact Free-Electron Laser (SACLA) (Ishikawa *et al.*, 2012 ▸). The sample-injection device stably extruded microcrystals embedded in the highly viscous carrier kept at a constant sample temperature.

## 2. Device for the injection of a highly viscous sample at atmospheric pressure   

### 2.1. High-viscosity cartridge-type injector   

**Figure 1:**
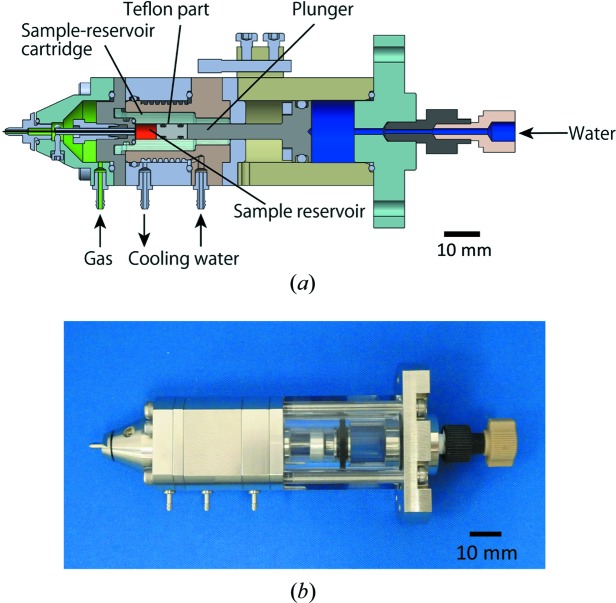
Schematic cross section (*a*) and photograph (*b*) of the HVC injector.

The HVC injector is designed to fit into the diffraction chamber of the Diverse Application Platform for Hard X-ray Diffraction in SACLA (DAPHNIS) (Tono *et al.*, 2015 ▸). Its structure and operation principle are based on the LCP injector developed by Weierstall *et al.* (2014 ▸). The HVC injector consists of a hydraulic cylinder, a sample reservoir, a cooling jacket and a nozzle (Fig. 1 ▸). An HPLC pump (LC-20AD, SHIMADZU) provides regulated water flow to the cylinder in order to apply the input pressure to a plunger in the hydraulic cylinder. The plunger pushes a highly viscous sample using a piston to continuously extrude it from an inner capillary in the nozzle. Typically, a hydraulic pressure of 0.2–0.4 MPa is applied for extruding LCP- or grease-embedded crystals with the use of a capillary nozzle having an inner diameter (ID) of 75 µm. The nozzle comprises an inner capillary nozzle (34 mm long) and an outer metallic nozzle for the co-flowing gas. The standard IDs of the inner nozzle are 75 and 100 µm, which are suitable for 5–30 µm microcrystals. Inner nozzles with different IDs (50, 125 and 150 µm) are also available for smaller or larger crystals. Each nozzle with a different ID has a common outer diameter (OD) of 360 µm. In all nozzles, the downstream end of the inner capillary is sharpened to a small taper angle (<10°) and protrudes from the outer nozzle by ∼500 µm to prevent the curling up of the extruded sample, as shown in Fig. S1 of the supporting information. Helium sheath gas is supplied from the outer nozzle to maintain a straight sample flow and is regulated by a flowmeter (FSM2-NVF050-H063N, CKD Corporation) placed outside the experimental hutch. Typically, the air-converted flow rate is between 0.3 and 0.5 l min^−1^ for all nozzles, depending on the type of highly viscous medium. The actual flow rate corrected for helium is approximately six times larger. Temperature-regulated water flows through the cooling jacket to keep the sample temperature constant. Although the injector is frequently subjected to high temperature (300–303 K) because of heat from the devices in the experimental hutch, the cooling water prevents the deterioration of the sample during data collection. Specifically, the LCP carrier is sensitive to temperature change and frequently changes its viscosity at higher temperature, which may result in droplet formation or instability of the sample stream. Thus, temperature control is essential for stable sample extrusion from the injector.

**Figure 2:**
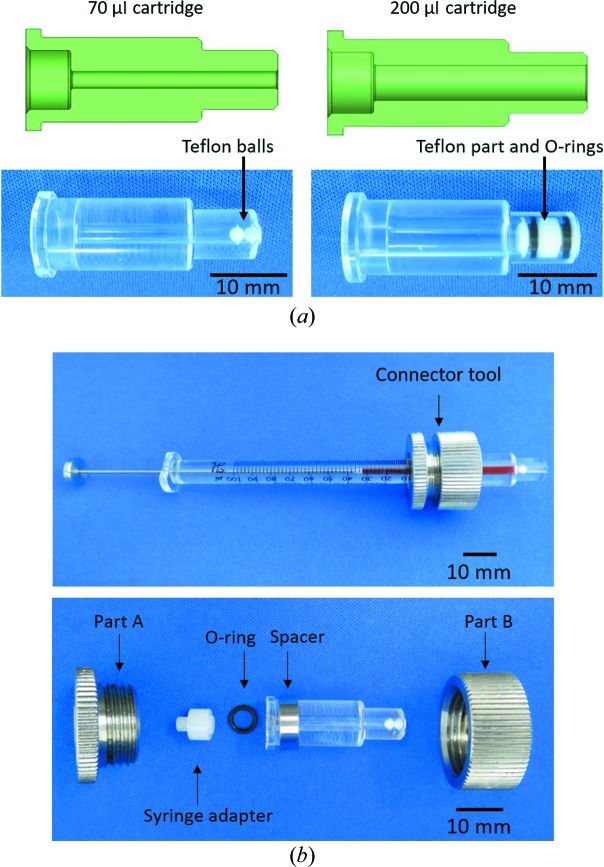
Cartridge-type sample reservoir. (*a*) Schematic cross-sectional view (top) and photograph of the cartridge with inserted Teflon parts (bottom) to form a tight seal. The two Teflon balls for the 70 µl cartridge reservoir have a diameter of 2 mm. The barrel-shaped part for the 200 µl cartridge reservoir consists of a Teflon part (8 mm in length, 4 mm in diameter) and two O-rings (OD of 4 m, ID of 2 mm). (*b*) Connector tool for sample loading from a Hamilton syringe into the sample reservoir. The connector tool consists of two metallic parts, a metallic ring spacer (OD of 12 mm, ID of 10 mm), an O-ring (OD of 6 mm, ID of 4.5 mm) and a Teflon syringe adapter (8 mm in length, 7 mm in diameter). The parts A and B are 11 mm long and 23 mm in diameter. Each part is assembled as shown in the bottom view, and then the Teflon balls are moved to the left end by a thin stick. Subsequently, a 100 µl gas-tight syringe (No. 81065, Hamilton) loaded with sample is connected to the tool. The sample is loaded into the cartridge from the syringe thorough the tool until the Teflon balls reach the right end. In the top view, grease colored with red ink was used as the sample. An underfilled sample reservoir is also available for sample injection by the HVC injector.

One of the most distinctive features of the HVC injector is a cartridge-type sample reservoir, which facilitates easy loading of the sample (Fig. 2 ▸). The cartridge reservoir is made of polymethyl methacrylate; this material was selected because of its chemical durability, high pressure resistance and transparency, which allows observation of the sample inside. Two types of cartridges, with an ID of 4 mm (internal volume of 200 µl) or 2 mm (internal volume of 70 µl), are used [Fig. 2 ▸(*a*)]. A Teflon plug, shown in Fig. 2 ▸(*a*), is employed to form a tight seal in the 200 µl cartridge. The barrel-shaped plug is 8 mm in length and 4 mm in diameter, and it has an O-ring (ID of 2 mm and OD of 4 mm) at each end. For the 70 µl cartridge, similar to the LCP injector, two Teflon balls are used to form a seal (Weierstall *et al.*, 2014 ▸). Acrylic cartridges can withstand a maximum hydraulic pressure of 1 MPa, which corresponds to 64 MPa on the inside sample in the 70 µl cartridge. A highly viscous sample in a 100 µl gas-tight syringe (No. 81065, Hamilton) can be loaded into the cartridge using the connector tool as shown in Fig. 2 ▸(*b*). Alternatively, a sample can be loaded through a needle connected to the syringe. After the syringe has been disconnected, the cartridge should be subjected to centrifugation (10–20 s, 8000*g*) to remove any air bubbles from the carrier medium, as shown in Fig. S2. The loaded sample settles into the downstream end of the cartridge by centrifugation and can be stored for later use if the cartridge is sealed with a flexible film such as Parafilm. Finally, the cartridge is inserted into the cooling jacket of the injector body, which is typically set to 293 K during measurements. The sample temperature inside the acrylic cartridge was kept at 293.2–293.4 K in the experimental hutch at 301 K using a water chiller set to 293 K. The sample temperature can be controlled from 277 to 313 K by changing the temperature of the circulating water.

In an SFX experiment using a nozzle with an ID of 75 µm, the sample flow rate is typically set to 0.24 µl min^−1^, which transports the sample 30 µm between XFEL pulses operated at 30 Hz. The minimum flow rate is approximately 0.1 µl min^−1^ depending on the crystal carrier. The injector can work continuously for 14 h with a 200 µl^−1^ sample cartridge. On the other hand, the optical laser pump and XFEL probe experiments require that a sufficient separation between the pump laser shots is maintained in order to avoid any light contamination. For example, photosystem II crystals mixed with grease were extruded at a flow rate of 5.6 µl min^−1^ from a nozzle with an ID of 150 µm for pump–probe measurements using nanosecond optical lasers. The largest ID nozzle was used to prevent clogging because the photosystem II crystals were as large as 100 µm and could diffract X-rays up to 2.1 Å. The high flow rate of 5.6 µl min^−1^ was necessary to avoid light contamination due to pump light scattering on the sample stream. In this case, the sample in the reservoir was consumed in 36 min (Suga *et al.*, 2017 ▸).

### 2.2. Sample suction device   

**Figure 3:**
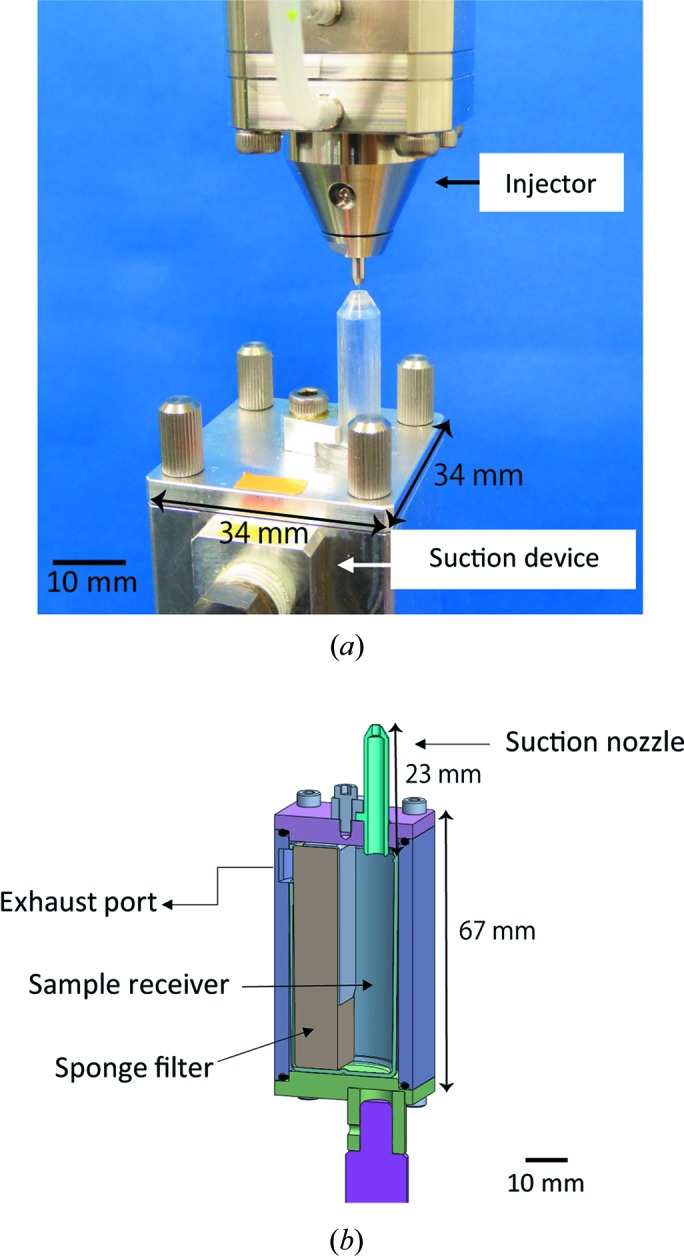
(*a*) Sample suction device mounted below the HVC injector. (*b*) Schematic cross-sectional view of the sample suction device. The suction nozzle has an OD of 4 mm and an ID of 2 mm and is tapered to prevent interference with diffraction from the crystals.

In addition to the helium sheath gas from the outer metallic nozzle, a sample suction device [Fig. 3 ▸(*a*)] is employed to maintain a straight sample stream from the nozzle of the injector. High-intensity XFEL pulses often chop a high-viscosity microstream; thereby, the sample is prone to curling up. The suction device prevents the curling up of samples, which is similar to the purpose of the sheath gas.

The suction device is composed of a suction tube, a sample receiver with a sponge filter and an exhaust port connected to a vacuum pump (DA-60S, ULVAC) with a maximum pumping speed of 72 l min^−1^, as shown in Fig. 3 ▸(*b*). The suction nozzle is placed approximately 1.8 mm directly below the injector nozzle for the purpose of sucking highly viscous samples after X-ray irradiation. The ID of the nozzle (2 mm) has been optimized to achieve a sufficient suction force. The suction device is connected to the vacuum pump outside the experimental hutch through a flexible vacuum hose (approximately 17 m long). A flowmeter (FSM2-NVF050-H063N, CKD Cor­pora­tion) is inserted in front of the vacuum pump to regulate the gas flow rate in the suction line from the outside of the hutch. Typically, the air-converted flow rate is 0.5–1 l min^−1^ (the actual flow rate of helium is 3–6 l min^−1^). Although the gas flow rate depends on the type of highly viscous medium, it is not affected by the IDs of the injector nozzles. The sample receiver for collecting the waste sample can be replaced easily. The sponge filter in the receiver prevents the suction line from clogging up with waste sample. The sample receiver has a capacity of ∼10 ml, but because the suction capability typically starts to degrade after 4–5 ml of the discarded samples have accumulated, the receiver should be exchanged once or twice in 24 h for SFX experiments requiring relatively high sample flow rates, *e.g.* 5.6 µl min^−1^.

## 3. Experimental   

### 3.1. Crystallization of A_2A_AR in complex with ZM241385   

A_2A_AR was engineered by replacement of the third intracellular loop with a thermostabilized apocytochrome b562, which was expressed using a baculovirus system in *Spodoptera frugiperda* Sf9 insect cells and then purified as described previously (Liu *et al.*, 2012 ▸; Nakane, Hanashima *et al.*, 2016 ▸). Crystals of A_2A_AR in complex with ZM241385 [4-(2-{7-amino-2-(2-furyl)[1,2,4]triazolo[2,3-*a*][1,3,5]triazin-5-yl­amino}­ethyl)­phenol] were obtained in LCP. Approximately 50 mg ml^−1^ of purified protein solution was mixed with a molten lipid using Hamilton syringes connected to a syringe coupler (Caffrey & Porter, 2010 ▸) and reconstituted in the LCP comprising 54%(*w*/*w*) monoolein (Sigma) and 6%(*w*/*w*) cholesterol (Avanti Polar Lipids). Crystals in LCP are typically grown in a syringe using the method developed by Liu *et al.* (2014 ▸) for SFX experiments. However, in this study, another crystallization method using a thin metal wire was applied. This method was originally developed for LCP-SFX (Nango *et al.*, 2016 ▸). Briefly, after reconstitution into LCP, the coupler and Teflon ferrule were removed, and a cleaning wire (∼2 cm long) was inserted into the protein-laden LCP inside the syringe. The protein-laden LCP was directly extruded from the syringe into a 0.6 ml Eppendorf tube, affording a column with a diameter of 1.5 mm, as shown in Fig. S3. The cylindrical LCP was immersed in a precipitant solution containing 27%(*v*/*v*) PEG 400, 50 m*M* sodium thio­cyanate, 2%(*v*/*v*) 2,5-hexane­diol and 100 m*M* sodium citrate (pH 5.0), and subsequently incubated at 293 K. A myriad of small crystals appeared within a week [Fig. S4(*a*)]. The wire helped to retain the cylindrical LCP. Before SFX measurements, monoolein was added to the LCP matrix for the absorption of the precipitant and its homogenization to a final concentration of ∼20%. The mixture was homogenized using two syringes connected to each other until it became transparent, loaded into a 70 µl sample cartridge of the HVC injector through a needle with an ID of 400 µm connected to one of the syringes, and then subjected to centrifugation at 2000*g* for several tens of seconds without the syringe.

### 3.2. Preparation of grease-dispersed lysozyme microcrystals   

Tetragonal hen egg-white lysozyme was crystallized by batch crystallization at 290 K as described previously (Sugahara *et al.*, 2015 ▸; Nango *et al.*, 2015 ▸). A 100 µl crystallization solution containing lysozyme microcrystals (2.3 × 10^8^ crystals ml^−1^) was subjected to centrifugation using a tabletop centrifuge for 10 s, followed by removal of 90 µl of the supernatant. Concentrated microcrystals were mixed with 90 µl of Super Lube nuclear grade grease (No. 42150, Synco Chemical Co.) on a plastic plate according to a previously reported procedure (Sugahara *et al.*, 2015 ▸). The crystals were directly transferred into a 200 µl sample cartridge using a spatula. After loading of the sample, the cartridge was subjected to centrifugation to remove bubbles in the mixture and then the cartridge was set into the HVC injector body.

### 3.3. SFX data collection   

Data were collected at BL3 of SACLA (Ishikawa *et al.*, 2012 ▸; Tono *et al.*, 2013 ▸) using 7.0 keV X-rays with a pulse duration of <10 fs and a repetition rate of 30 Hz. The XFEL beam was focused on a beam diameter of 1.5 µm FWHM with two elliptical mirrors in the Kirkpatrick–Baez geometry. The number of X-ray photons at the sample position was ∼2 × 10^11^ photons per pulse for the A_2A_AR crystals. For the lysozyme crystals, the beam was attenuated to have ∼6 × 10^10^ photons per pulse to avoid saturation of the detector. The HVC injector was installed in an He-gas-filled diffraction chamber of the DAPHNIS setup (Tono *et al.*, 2015 ▸). The sample was maintained at 293 K using the cooling jacket of the injector, although the chamber temperature was relatively high (299–300 K). The suction device was placed immediately below the injector. The helium gas aspirated by the suction device was returned from the exhaust line of the vacuum pump to the chamber. Diffraction patterns were recorded using a multiport CCD detector with eight sensor modules (Kameshima *et al.*, 2014 ▸) at a sample-to-detector distance of 50 mm. A_2A_AR crystals (20 × 3 × 3 µm) in an LCP matrix were extruded at a flow rate of 0.24 µl min^−1^ from a nozzle of 75 µm ID. Grease-embedded lysozyme crystals (∼5 µm in size) were ejected at a flow rate of 0.42 µl min^−1^ from a nozzle with an ID of 100 µm.

### 3.4. Data processing and structure determination   

Diffraction images were filtered to extract ‘hit’ images using the *Cheetah* software package (Barty *et al.*, 2014 ▸), which was modified for the SFX data processing at SACLA (Nakane, Joti *et al.*, 2016 ▸). A hit image was defined as an image containing a minimum of 20 Bragg peaks. The detector geometry was refined by *geoptimiser* in *CrystFEL* (version 0.6.3; White *et al.*, 2013 ▸, 2016 ▸); auto-indexing and integration were performed using *CrystFEL* and *DirAx* (Duisenberg, 1992 ▸). The resolution cutoffs were assumed to be 2.25 Å for A_2A_AR and 2.0 Å for the lysozyme from CC_1/2_ and *R*_split_.

**Figure 4:**
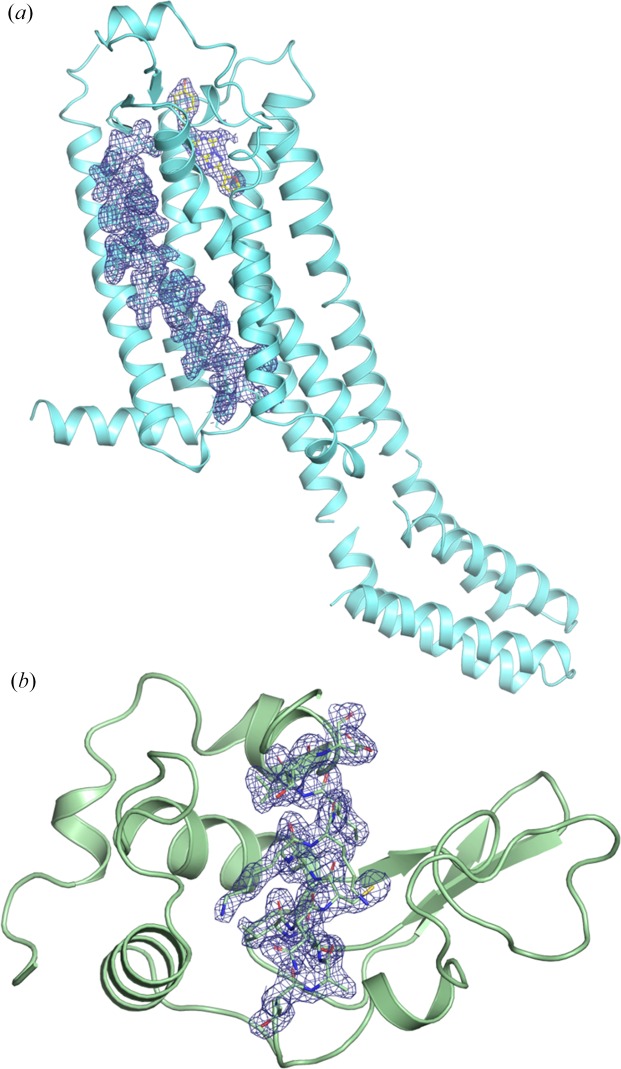
(*a*) Structure of human A_2A_ adenosine receptor complexed with ZM241385, showing 2*F*_o_−*F*_c_ density (blue, contoured at 1.0σ). The antagonist, ZM241385, is shown as a stick model with yellow carbon atoms. The residues between 41 and 66 are shown as a stick model with cyan carbon atoms. The electron-density map covers the residues and ZM241385. (*b*) Structure of hen egg-white lysozyme, showing the 2*F*_o_−*F*_c_ density (blue, contoured at 1.0σ) corresponding to the residues between 89 and 101.

The structure determination of A_2A_AR was performed by molecular replacement with the program *MOLREP* (Vagin & Teplyakov, 2010 ▸), which is a part of the *CCP4* software package, using the A_2A_AR structure (PDB ID 4eiy; Liu *et al.*, 2012 ▸) as the search model. The lysozyme structure was solved by rigid-body refinement using 5wrb (Sugahara *et al.*, 2017 ▸) with the *Phenix* suite (Adams *et al.*, 2002 ▸). All refinements were performed using *phenix.refine* (Afonine *et al.*, 2012 ▸) followed by the manual examination and rebuilding of the refined coordinates in *Coot* (Emsley *et al.*, 2010 ▸), which results in *R*_work_/*R*_free_ = 18.3/21.8 (A_2A_AR) and *R*_work_/*R*_free_ = 15.9/20.2 (lysozyme). The data collection and refinement statistics are summarized in Table 1 ▸. Fig. 4 ▸ was created using *PyMOL* (DeLano Scientific; http://www.pymol.org).

## 4. Results   

A_2A_AR complexed with ZM241385 was crystallized with average dimensions of 20 × 3 × 3 µm in LCP [Fig. S4(*a*)]. A total of 190 041 diffraction patterns were collected from *ca* 30 µl of the A_2A_AR crystals embedded in LCP, of which 10 816 were identified as hit images, corresponding to an average hit rate of 5.7%. The hit rate was relatively low because of the low crystal density [Fig. S4(*a*)], which is common during LCP crystallization. The remaining images showed weak scattering patterns from lipids without strong diffraction rings (Fig. S5). Of the hit images, 10 510 patterns were indexed. The indexing rate was as high as 97%. Although the sample stream was chopped by the XFEL beam during data collection, the suction device and a co-flowing sheath gas delivered samples to the interaction point without coiling up. Movie S1 provided in the supporting information shows a sample stream from the HVC injector during data collection. The HVC injector with the cooling jacket successfully prevented any sample deterioration, including viscosity change due to phase transitions resulting from changes in temperature, allowing us to keep an almost constant hit rate during the 114 min of data collection. In general, crystals in LCP are sensitive to temperature change, which can result in crystal dissolution. Moreover, the sample injection under atmospheric pressure did not require the addition of 7.9 MAG or 9.7 MAG to the LCP to prevent the transition to lamellar crystalline phases due to evaporative cooling (Liu *et al.*, 2014 ▸). The structure of A_2A_AR complexed with ZM241385 was solved at a resolution of 2.25 Å using 10 510 indexed images [Fig. 4 ▸(*a*)].

A full data set was also collected for the grease-dispersed lysozyme crystals (∼5 µm in size). A total of 94 061 diffraction patterns were obtained from *ca* 40 µl of sample. The hit and indexing rates were 43.5 and 70.3%, respectively, that is, 28 779 patterns were indexed (Table 1 ▸). The hit rate was high because of the high concentration of lysozyme crystals [Fig. S4(*b*)]. The indexing rate was relatively low as the diffraction patterns from multiple crystals were frequently recorded in a single image because of the high crystal density. Data were collected continuously for 54 min without the nozzle clogging or the need for sample replacement. The lysozyme structure was determined at a resolution of 2.0 Å [Fig. 4 ▸(*b*)].

## 5. Discussion   

Serial crystallography using highly viscous samples at synchrotron X-ray sources has been reported previously (Botha *et al.*, 2015 ▸). Highly viscous microstreams were guided by helium sheath gas and delivered into an X-ray beam interaction point under atmospheric pressure. A catcher connected to a sucking fan served only to collect the extruded samples. In the case of XFEL experiments, intense X-ray pulses give rise to shock waves in a fluid sample (Stan *et al.*, 2016 ▸), which disturbs the stable stream of the sample. In addition, highly viscous carrier media are adhesive, causing coiling around a nozzle once the extrusion is disturbed. In the device described herein, in addition to the helium sheath gas, we employ the suction device to assist sample injection under atmospheric pressure, resulting in a stable sample stream during data collection.

The HVC injector has been applied to other protein crystals, allowing for the structure determination of the human orexin-2 receptor (Suno *et al.*, 2018 ▸), the M2 proton channel of influenza A (Thomaston *et al.*, 2017 ▸), bacterial phytochrome (Edlund *et al.*, 2016 ▸), cytochrome c oxidase (Andersson *et al.*, 2017 ▸) and photosystem II (Suga *et al.*, 2017 ▸). The HVC injector is applicable not only to LCP and grease but also to other carrier media, such as hy­droxy­ethyl cellulose (Sugahara *et al.*, 2017 ▸; Tosha *et al.*, 2017 ▸). The successful results using the injector from SFX experiments are summarized in Table S1 of the supporting information.

The HVC injector has also been successfully used for determining a dynamic protein structure from time-resolved SFX data (Table S1; Nango *et al.*, 2016 ▸; Sugahara *et al.*, 2017 ▸; Tosha *et al.*, 2017 ▸). The first pump–probe SFX experiment using the HVC injector was performed using bacteriorhodopsin crystallized in LCP at SACLA (Nango *et al.*, 2016 ▸). In the experiment, pump laser light was scattered by the sample streams, which caused light contamination in the bacteriorhodopsin crystals. Therefore, a faster sample flow was crucial to secure enough separation between illuminated points of the sample to avoid light contamination. The injector was able to maintain a stable sample injection even at a high flow rate of 2.5 µl min^−1^. Although sample exchange was needed once or twice per hour owing to the high sample consumption, it could be done smoothly since the sample cartridge facilitated sample loading. Consequently, full data sets from bacteriorhodopsin crystals could be collected at 13 time points, from nanoseconds to milliseconds, following light irradiation in 48 h of beam time.

## 6. Summary   

Our injection device for highly viscous samples enables the stable extrusion of crystals embedded in various carrier media in SFX experiments under atmospheric pressure. The sample reservoir of the injector is a transparent cartridge, allowing samples to be loaded easily and checked visually. The injector can be readily assembled and mounted in a diffraction chamber. This means that the injector system is highly compatible with the atmospheric pressure SFX instruments at other XFEL/synchrotron-radiation facilities, where the necessary components for injector operation are generally available (*e.g.* an HPLC pump, a vacuum pump, standard piping components and regulated helium gas supply). The sample temperature is controlled at a constant value by a cooling jacket on the injector to avoid altering the sample properties. The suction device is of great use for stable sample injection as well as for collecting the waste from samples. The injection device has been applied to a wide variety of proteins, achieving structure determination of proteins including dynamic structures by time-resolved SFX.

## Supplementary Material

A sample stream from the HVC injector during data collection. DOI: 10.1107/S1600576719012846/te5044sup1.mp4

Supporting figures and table. DOI: 10.1107/S1600576719012846/te5044sup2.pdf

PDB reference: 6jzh

PDB reference: 6jzi

## Figures and Tables

**Table 1:** Crystallographic statistics Values in parentheses are those of the highest-resolution shell.

	A_2 A_AR/ZM241385	Lysozyme
Data collection		
Carrier	Monoolein mixture	Grease
Resolution range (Å)	44.90–2.25 (2.29–2.25)	39.6–2.00 (2.03–2.00)
Space group	*C*222_1_	*P*4_3_2_1_2
Unit-cell parameters		
*a* (Å)	40.2	79.1
*b* (Å)	179.6	79.1
*c* (Å)	142.1	38.0
No. of collected images	190 041	94 061
No. of hits	10 816	40 962
No. of indexed images	10 510	28 779
Indexing rate from hits (%)	97.2	43.5
No. of total reflections	3 487 348	5 059 907
No. of unique reflections	25 057	8615
Completeness (%)	100 (100)	100 (100)
Multiplicity	139 (71.9)	587 (82.6)
*R*_split_ (%)†	13.8 (37.4)	5.3 (21.3)
CC_1/2_ (%)	97.0 (81.1)	99.5 (90.3)
〈*I*/σ(*I*)〉	5.9 (2.5)	16.8 (4.7)
		
Refinement		
*R*/*R*_free_ (%)	18.3/21.8	15.9/20.2
R.m.s. deviation from ideal		
Bond lengths (Å)	0.003	0.005
Bond angles (°)	0.652	0.746
PDB code	6jzh	6jzi

†
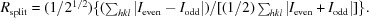

## References

[bb1] Adams, P. D., Grosse-Kunstleve, R. W., Hung, L.-W., Ioerger, T. R., McCoy, A. J., Moriarty, N. W., Read, R. J., Sacchettini, J. C., Sauter, N. K. & Terwilliger, T. C. (2002). *Acta Cryst.* D**58**, 1948–1954. 10.1107/s0907444902016657 12393927

[bb3] Afonine, P. V., Grosse-Kunstleve, R. W., Echols, N., Headd, J. J., Moriarty, N. W., Mustyakimov, M., Terwilliger, T. C., Urzhumtsev, A., Zwart, P. H. & Adams, P. D. (2012). *Acta Cryst.* D**68**, 352–367. 10.1107/S0907444912001308 PMC3322595 22505256

[bb5] Andersson, R., Safari, C., Dods, R., Nango, E., Tanaka, R., Yamashita, A., Nakane, T., Tono, K., Joti, Y., Båth, P., Dunevall, E., Bosman, R., Nureki, O., Iwata, S., Neutze, R. & Brändén, G. (2017). *Sci. Rep.* **7**, 4518. 10.1038/s41598-017-04817-z PMC5495810 28674417

[bb7] Barty, A., Kirian, R. A., Maia, F. R. N. C., Hantke, M., Yoon, C. H., White, T. A. & Chapman, H. (2014). *J. Appl. Cryst.* **47**, 1118–1131. 10.1107/S1600576714007626 PMC4038800 24904246

[bb9] Botha, S., Nass, K., Barends, T. R. M., Kabsch, W., Latz, B., Dworkowski, F., Foucar, L., Panepucci, E., Wang, M., Shoeman, R. L., Schlichting, I. & Doak, R. B. (2015). *Acta Cryst.* D**71**, 387–397. 10.1107/S1399004714026327 25664750

[bb11] Boutet, S., Lomb, L., Williams, G. J., Barends, T. R., Aquila, A., Doak, R. B., Weierstall, U., DePonte, D. P., Steinbrener, J., Shoeman, R. L., Messerschmidt, M., Barty, A., White, T. A., Kassemeyer, S., Kirian, R. A., Seibert, M. M., Montanez, P. A., Kenney, C., Herbst, R., Hart, P., Pines, J., Haller, G., Gruner, S. M., Philipp, H. T., Tate, M. W., Hromalik, M., Koerner, L. J., van Bakel, N., Morse, J., Ghonsalves, W., Arnlund, D., Bogan, M. J., Caleman, C., Fromme, R., Hampton, C. Y., Hunter, M. S., Johansson, L. C., Katona, G., Kupitz, C., Liang, M., Martin, A. V., Nass, K., Redecke, L., Stellato, F., Timneanu, N., Wang, D., Zatsepin, N. A., Schafer, D., Defever, J., Neutze, R., Fromme, P., Spence, J. C., Chapman, H. N. & Schlichting, I. (2012). *Science*, **337**, 362–364.

[bb13] Caffrey, M. & Cherezov, V. (2009). *Nat. Protoc.* **4**, 706–731. 10.1038/nprot.2009.31 PMC2732203 19390528

[bb15] Caffrey, M. & Porter, C. (2010). *J. Vis. Exp.* **21**, 1712. 10.3791/1712 PMC3144658 21113125

[bb17] Chapman, H. N., Fromme, P., Barty, A., White, T. A., Kirian, R. A., Aquila, A., Hunter, M. S., Schulz, J., DePonte, D. P., Weierstall, U., Doak, R. B., Maia, F. R., Martin, A. V., Schlichting, I., Lomb, L., Coppola, N., Shoeman, R. L., Epp, S. W., Hartmann, R., Rolles, D., Rudenko, A., Foucar, L., Kimmel, N., Weidenspointner, G., Holl, P., Liang, M., Barthelmess, M., Caleman, C., Boutet, S., Bogan, M. J., Krzywinski, J., Bostedt, C., Bajt, S., Gumprecht, L., Rudek, B., Erk, B., Schmidt, C., Hömke, A., Reich, C., Pietschner, D., Strüder, L., Hauser, G., Gorke, H., Ullrich, J., Herrmann, S., Schaller, G., Schopper, F., Soltau, H., Kühnel, K. U., Messerschmidt, M., Bozek, J. D., Hau-Riege, S. P., Frank, M., Hampton, C. Y., Sierra, R. G., Starodub, D., Williams, G. J., Hajdu, J., Timneanu, N., Seibert, M. M., Andreasson, J., Rocker, A., Jönsson, O., Svenda, M., Stern, S., Nass, K., Andritschke, R., Schröter, C. D., Krasniqi, F., Bott, M., Schmidt, K. E., Wang, X., Grotjohann, I., Holton, J. M., Barends, T. R., Neutze, R., Marchesini, S., Fromme, R., Schorb, S., Rupp, D., Adolph, M., Gorkhover, T., Andersson, I., Hirsemann, H., Potdevin, G., Graafsma, H., Nilsson, B. & Spence, J. C. (2011). *Nature*, **470**, 73–77.

[bb19] Conrad, C. E., Basu, S., James, D., Wang, D., Schaffer, A., Roy-Chowdhury, S., Zatsepin, N. A., Aquila, A., Coe, J., Gati, C., Hunter, M. S., Koglin, J. E., Kupitz, C., Nelson, G., Subramanian, G., White, T. A., Zhao, Y., Zook, J., Boutet, S., Cherezov, V., Spence, J. C. H., Fromme, R., Weierstall, U. & Fromme, P. (2015). *IUCrJ*, **2**, 421–430. 10.1107/S2052252515009811 PMC4491314 26177184

[bb21] DePonte, D. P., Weierstall, U., Schmidt, K., Warner, J., Starodub, D., Spence, J. C. H. & Doak, R. B. (2008). *J. Phys. D Appl. Phys.* **41**, 195505.

[bb81] Duisenberg, A. J. M. (1992). *J. Appl. Cryst.* **25**, 92–96.

[bb23] Edlund, P., Takala, H., Claesson, E., Henry, L., Dods, R., Lehtivuori, H., Panman, M., Pande, K., White, T., Nakane, T., Berntsson, O., Gustavsson, E., Båth, P., Modi, V., Roy-Chowdhury, S., Zook, J., Berntsen, P., Pandey, S., Poudyal, I., Tenboer, J., Kupitz, C., Barty, A., Fromme, P., Koralek, J. D., Tanaka, T., Spence, J., Liang, M., Hunter, M. S., Boutet, S., Nango, E., Moffat, K., Groenhof, G., Ihalainen, J., Stojković, E. A., Schmidt, M. & Westenhoff, S. (2016). *Sci. Rep.* **6**, 35279. 10.1038/srep35279 PMC5069500 27756898

[bb25] Emsley, P., Lohkamp, B., Scott, W. G. & Cowtan, K. (2010). *Acta Cryst.* D**66**, 486–501. 10.1107/S0907444910007493 PMC2852313 20383002

[bb27] Ishikawa, T., Aoyagi, H., Asaka, T., Asano, Y., Azumi, N., Bizen, T., Ego, H., Fukami, K., Fukui, T., Furukawa, Y., Goto, S., Hanaki, H., Hara, T., Hasegawa, T., Hatsui, T., Higashiya, A., Hirono, T., Hosoda, N., Ishii, M., Inagaki, T., Inubushi, Y., Itoga, T., Joti, Y., Kago, M., Kameshima, T., Kimura, H., Kirihara, Y., Kiyomichi, A., Kobayashi, T., Kondo, C., Kudo, T., Maesaka, H., Marechal, X. M., Masuda, T., Matsubara, S., Matsumoto, T., Matsushita, T., Matsui, S., Nagasono, M., Nariyama, N., Ohashi, H., Ohata, T., Ohshima, T., Ono, S., Otake, Y., Saji, C., Sakurai, T., Sato, T., Sawada, K., Seike, T., Shirasawa, K., Sugimoto, T., Suzuki, S., Takahashi, S., Takebe, H., Takeshita, K., Tamasaku, K., Tanaka, H., Tanaka, R., Tanaka, T., Togashi, T., Togawa, K., Tokuhisa, A., Tomizawa, H., Tono, K., Wu, S. K., Yabashi, M., Yamaga, M., Yamashita, A., Yanagida, K., Zhang, C., Shintake, T., Kitamura, H. & Kumagai, N. (2012). *Nat. Photon.*, **6**, 540–544.

[bb29] Kameshima, T., Ono, S., Kudo, T., Ozaki, K., Kirihara, Y., Kobayashi, K., Inubushi, Y., Yabashi, M., Horigome, T., Holland, A., Holland, K., Burt, D., Murao, H. & Hatsui, T. (2014). *Rev. Sci. Instrum.* **85**, 033110. 10.1063/1.4867668 24689567

[bb31] Kovácsová, G., Grünbein, M. L., Kloos, M., Barends, T. R. M., Schlesinger, R., Heberle, J., Kabsch, W., Shoeman, R. L., Doak, R. B. & Schlichting, I. (2017). *IUCrJ*, **4**, 400–410. 10.1107/S2052252517005140 PMC5571803 28875027

[bb33] Landau, E. M. & Rosenbusch, J. P. (1996). *Proc. Natl Acad. Sci. USA*, **93**, 14532–14535. 10.1073/pnas.93.25.14532 PMC26167 8962086

[bb35] Liu, W., Chun, E., Thompson, A. A., Chubukov, P., Xu, F., Katritch, V., Han, G. W., Roth, C. B., Heitman, L. H., IJzerman, A. P., Cherezov, V. & Stevens, R. C. (2012). *Science*, **337**, 232–236. 10.1126/science.1219218 PMC3399762 22798613

[bb37] Liu, W., Ishchenko, A. & Cherezov, V. (2014). *Nat. Protoc.* **9**, 2123–2134. 10.1038/nprot.2014.141 PMC4209290 25122522

[bb39] Martin-Garcia, J. M., Conrad, C. E., Nelson, G., Stander, N., Zatsepin, N. A., Zook, J., Zhu, L., Geiger, J., Chun, E., Kissick, D., Hilgart, M. C., Ogata, C., Ishchenko, A., Nagaratnam, N., Roy-Chowdhury, S., Coe, J., Subramanian, G., Schaffer, A., James, D., Ketwala, G., Venugopalan, N., Xu, S., Corcoran, S., Ferguson, D., Weierstall, U., Spence, J. C. H., Cherezov, V., Fromme, P., Fischetti, R. F. & Liu, W. (2017). *IUCrJ*, **4**, 439–454. 10.1107/S205225251700570X PMC5571807 28875031

[bb41] Nakane, T., Hanashima, S., Suzuki, M., Saiki, H., Hayashi, T., Kakinouchi, K., Sugiyama, S., Kawatake, S., Matsuoka, S., Matsumori, N., Nango, E., Kobayashi, J., Shimamura, T., Kimura, K., Mori, C., Kunishima, N., Sugahara, M., Takakyu, Y., Inoue, S., Masuda, T., Hosaka, T., Tono, K., Joti, Y., Kameshima, T., Hatsui, T., Yabashi, M., Inoue, T., Nureki, O., Iwata, S., Murata, M. & Mizohata, E. (2016). *Proc. Natl Acad. Sci. USA*, **113**, 13039–13044. 10.1073/pnas.1602531113 PMC5135358 27799539

[bb43] Nakane, T., Joti, Y., Tono, K., Yabashi, M., Nango, E., Iwata, S., Ishitani, R. & Nureki, O. (2016). *J. Appl. Cryst.* **49**, 1035–1041. 10.1107/S1600576716005720 PMC4886989 27275146

[bb45] Nango, E., Royant, A., Kubo, M., Nakane, T., Wickstrand, C., Kimura, T., Tanaka, T., Tono, K., Song, C., Tanaka, R., Arima, T., Yamashita, A., Kobayashi, J., Hosaka, T., Mizohata, E., Nogly, P., Sugahara, M., Nam, D., Nomura, T., Shimamura, T., Im, D., Fujiwara, T., Yamanaka, Y., Jeon, B., Nishizawa, T., Oda, K., Fukuda, M., Andersson, R., Båth, P., Dods, R., Davidsson, J., Matsuoka, S., Kawatake, S., Murata, M., Nureki, O., Owada, S., Kameshima, T., Hatsui, T., Joti, Y., Schertler, G., Yabashi, M., Bondar, A. N., Standfuss, J., Neutze, R. & Iwata, S. (2016). *Science*, **354**, 1552–1557. 10.1126/science.aah3497 28008064

[bb47] Nango, E., Sugahara, M., Kobayashi, J., Tanaka, T., Yamashita, A., Pan, D., Tanaka, Y., Ihara, K., Suno, C. & Shimamura, T. (2015). *PSSJ Archives*, **8**, e081.

[bb49] Neutze, R., Wouts, R., van der Spoel, D., Weckert, E. & Hajdu, J. (2000). *Nature*, **406**, 752–757. 10.1038/35021099 10963603

[bb51] Sierra, R. G., Batyuk, A., Sun, Z., Aquila, A., Hunter, M. S., Lane, T. J., Liang, M., Yoon, C. H., Alonso-Mori, R., Armenta, R., Castagna, J.-C., Hollenbeck, M., Osier, T. O., Hayes, M., Aldrich, J., Curtis, R., Koglin, J. E., Rendahl, T., Rodriguez, E., Carbajo, S., Guillet, S., Paul, R., Hart, P., Nakahara, K., Carini, G., DeMirci, H., Dao, E. H., Hayes, B. M., Rao, Y. P., Chollet, M., Feng, Y., Fuller, F. D., Kupitz, C., Sato, T., Seaberg, M. H., Song, S., van Driel, T. B., Yavas, H., Zhu, D., Cohen, A. E., Wakatsuki, S. & Boutet, S. (2019). *J. Synchrotron Rad.* **26**, 346–357. 10.1107/S1600577519001577 PMC6412173 30855242

[bb53] Sierra, R. G., Laksmono, H., Kern, J., Tran, R., Hattne, J., Alonso-Mori, R., Lassalle-Kaiser, B., Glöckner, C., Hellmich, J., Schafer, D. W., Echols, N., Gildea, R. J., Grosse-Kunstleve, R. W., Sellberg, J., McQueen, T. A., Fry, A. R., Messerschmidt, M. M., Miahnahri, A., Seibert, M. M., Hampton, C. Y., Starodub, D., Loh, N. D., Sokaras, D., Weng, T.-C., Zwart, P. H., Glatzel, P., Milathianaki, D., White, W. E., Adams, P. D., Williams, G. J., Boutet, S., Zouni, A., Messinger, J., Sauter, N. K., Bergmann, U., Yano, J., Yachandra, V. K. & Bogan, M. J. (2012). *Acta Cryst.* D**68**, 1584–1587.

[bb55] Stan, C. A., Milathianaki, D., Laksmono, H., Sierra, R. G., McQueen, T. A., Messerschmidt, M., Williams, G. J., Koglin, J. E., Lane, T. J., Hayes, M. J., Guillet, S. A. H., Liang, M. N., Aquila, A. L., Willmott, P. R., Robinson, J. S., Gumerlock, K. L., Botha, S., Nass, K., Schlichting, I., Shoeman, R. L., Stone, H. A. & Boutet, S. (2016). *Nat. Phys.* **12**, 966–971.

[bb57] Suga, M., Akita, F., Sugahara, M., Kubo, M., Nakajima, Y., Nakane, T., Yamashita, K., Umena, Y., Nakabayashi, M., Yamane, T., Nakano, T., Suzuki, M., Masuda, T., Inoue, S., Kimura, T., Nomura, T., Yonekura, S., Yu, L. J., Sakamoto, T., Motomura, T., Chen, J. H., Kato, Y., Noguchi, T., Tono, K., Joti, Y., Kameshima, T., Hatsui, T., Nango, E., Tanaka, R., Naitow, H., Matsuura, Y., Yamashita, A., Yamamoto, M., Nureki, O., Yabashi, M., Ishikawa, T., Iwata, S. & Shen, J. R. (2017). *Nature*, **543**, 131–135.

[bb59] Sugahara, M., Mizohata, E., Nango, E., Suzuki, M., Tanaka, T., Masuda, T., Tanaka, R., Shimamura, T., Tanaka, Y., Suno, C., Ihara, K., Pan, D., Kakinouchi, K., Sugiyama, S., Murata, M., Inoue, T., Tono, K., Song, C., Park, J., Kameshima, T., Hatsui, T., Joti, Y., Yabashi, M. & Iwata, S. (2015). *Nat. Methods*, **12**, 61–63. 10.1038/nmeth.3172 25384243

[bb61] Sugahara, M., Nakane, T., Masuda, T., Suzuki, M., Inoue, S., Song, C., Tanaka, R., Nakatsu, T., Mizohata, E., Yumoto, F., Tono, K., Joti, Y., Kameshima, T., Hatsui, T., Yabashi, M., Nureki, O., Numata, K., Nango, E. & Iwata, S. (2017). *Sci. Rep.* **7**, 703. 10.1038/s41598-017-00761-0 PMC5429652 28386083

[bb63] Sugahara, M., Song, C., Suzuki, M., Masuda, T., Inoue, S., Nakane, T., Yumoto, F., Nango, E., Tanaka, R., Tono, K., Joti, Y., Kameshima, T., Hatsui, T., Yabashi, M., Nureki, O., Numata, K. & Iwata, S. (2016). *Sci. Rep.* **6**, 24484. 10.1038/srep24484 PMC4834484 27087008

[bb65] Suno, R., Kimura, K. T., Nakane, T., Yamashita, K., Wang, J., Fujiwara, T., Yamanaka, Y., Im, D., Horita, S., Tsujimoto, H., Tawaramoto, M. S., Hirokawa, T., Nango, E., Tono, K., Kameshima, T., Hatsui, T., Joti, Y., Yabashi, M., Shimamoto, K., Yamamoto, M., Rosenbaum, D. M., Iwata, S., Shimamura, T. & Kobayashi, T. (2018). *Structure*, **26**, 7–19. 10.1016/j.str.2017.11.005 29225076

[bb67] Thomaston, J. L., Woldeyes, R. A., Nakane, T., Yamashita, A., Tanaka, T., Koiwai, K., Brewster, A. S., Barad, B. A., Chen, Y., Lemmin, T., Uervirojnangkoorn, M., Arima, T., Kobayashi, J., Masuda, T., Suzuki, M., Sugahara, M., Sauter, N. K., Tanaka, R., Nureki, O., Tono, K., Joti, Y., Nango, E., Iwata, S., Yumoto, F., Fraser, J. S. & DeGrado, W. F. (2017). *Proc. Natl Acad. Sci. USA*, **114**, 13357–13362. 10.1073/pnas.1705624114 PMC5754760 28835537

[bb69] Tono, K., Nango, E., Sugahara, M., Song, C., Park, J., Tanaka, T., Tanaka, R., Joti, Y., Kameshima, T., Ono, S., Hatsui, T., Mizohata, E., Suzuki, M., Shimamura, T., Tanaka, Y., Iwata, S. & Yabashi, M. (2015). *J. Synchrotron Rad.* **22**, 532–537. 10.1107/S1600577515004464 PMC4817517 25931065

[bb71] Tono, K., Togashi, T., Inubushi, Y., Sato, T., Katayama, T., Ogawa, K., Ohashi, H., Kimura, H., Takahashi, S., Takeshita, K., Tomizawa, H., Goto, S., Ishikawa, T. & Yabashi, M. (2013). *New J. Phys.* **15**, 083035.

[bb73] Tosha, T., Nomura, T., Nishida, T., Saeki, N., Okubayashi, K., Yamagiwa, R., Sugahara, M., Nakane, T., Yamashita, K., Hirata, K., Ueno, G., Kimura, T., Hisano, T., Muramoto, K., Sawai, H., Takeda, H., Mizohata, E., Yamashita, A., Kanematsu, Y., Takano, Y., Nango, E., Tanaka, R., Nureki, O., Shoji, O., Ikemoto, Y., Murakami, H., Owada, S., Tono, K., Yabashi, M., Yamamoto, M., Ago, H., Iwata, S., Sugimoto, H., Shiro, Y. & Kubo, M. (2017). *Nat. Commun.* **8**, 1585. 10.1038/s41467-017-01702-1 PMC5691058 29147002

[bb75] Vagin, A. & Teplyakov, A. (2010). *Acta Cryst.* D**66**, 22–25. 10.1107/S0907444909042589 20057045

[bb77] Weierstall, U., James, D., Wang, C., White, T. A., Wang, D., Liu, W., Spence, J. C., Bruce Doak, R., Nelson, G., Fromme, P., Fromme, R., Grotjohann, I., Kupitz, C., Zatsepin, N. A., Liu, H., Basu, S., Wacker, D., Won Han, G., Katritch, V., Boutet, S., Messerschmidt, M., Williams, G. J., Koglin, J. E., Marvin Seibert, M., Klinker, M., Gati, C., Shoeman, R. L., Barty, A., Chapman, H. N., Kirian, R. A., Beyerlein, K. R., Stevens, R. C., Li, D., Shah, S. T., Howe, N., Caffrey, M. & Cherezov, V. (2014). *Nat. Commun.* **5**, 3309. 10.1038/ncomms4309 PMC4061911 24525480

[bb79] White, T. A., Barty, A., Stellato, F., Holton, J. M., Kirian, R. A., Zatsepin, N. A. & Chapman, H. N. (2013). *Acta Cryst.* D**69**, 1231–1240. 10.1107/S0907444913013620 PMC3689526 23793149

[bb80] White, T. A., Mariani, V., Brehm, W., Yefanov, O., Barty, A., Beyerlein, K. R., Chervinskii, F., Galli, L., Gati, C., Nakane, T., Tolstikova, A., Yamashita, K., Yoon, C. H., Diederichs, K. & Chapman, H. N. (2016). *J. Appl. Cryst.* **49**, 680–689. 10.1107/S1600576716004751 PMC4815879 27047311

