# Supplementary material for: High-viscosity sample-injection device for serial femtosecond crystallography at atmospheric pressure
Source: J Appl Crystallogr. 2019 Oct 17;52(Pt 6):1280–8. doi: 10.1107/S1600576719012846 (PMC6878880; doi:10.1107/S1600576719012846)
Supplement: Supplementary file 2 [file j-52-01280-sup2.pdf]

Appl Cryst JAC JOURNAL OF APPLIED CRYSTALLOGRAPHY

Volume 52 (2019)

Supporting information for article:

High-viscosity sample injection device for serial femtosecond crystallography at atmospheric pressure High-viscosity sample injection device for serial femtosecond crystallography at atmospheric pressure

Yoshiaki Shimazu, Kensuke Tono, Tomoyuki Tanaka, Yasuaki Yamanaka, Takanori Nakane, Chihiro Mori, Kanako Terakado Kimura, Takaaki Fujiwara, Michihiro Sugahara, Rie Tanaka, R. Bruce Doak, Tatsuro Shimamura, So Iwata, Eriko Nango and Makina Yabashi

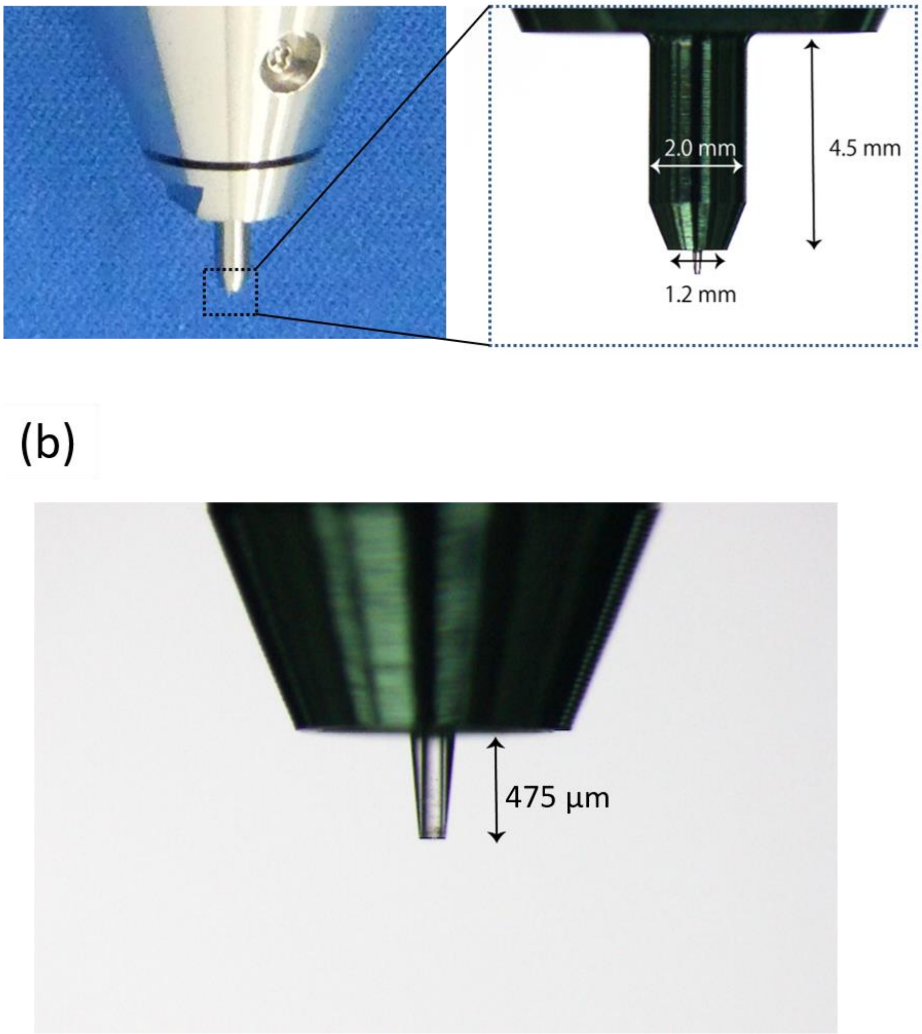

Figure S1 Images of the injector nozzle. (a) Magnified view of an outer metallic nozzle for a sheath gas of the HVE injector. The OD of the outer nozzle is 1.2 mm. (b) Magnified view of the capillary nozzle tip. The capillary is sharpened to a small taper angle (<10°) and protrudes from the outer nozzle by 475 µm.

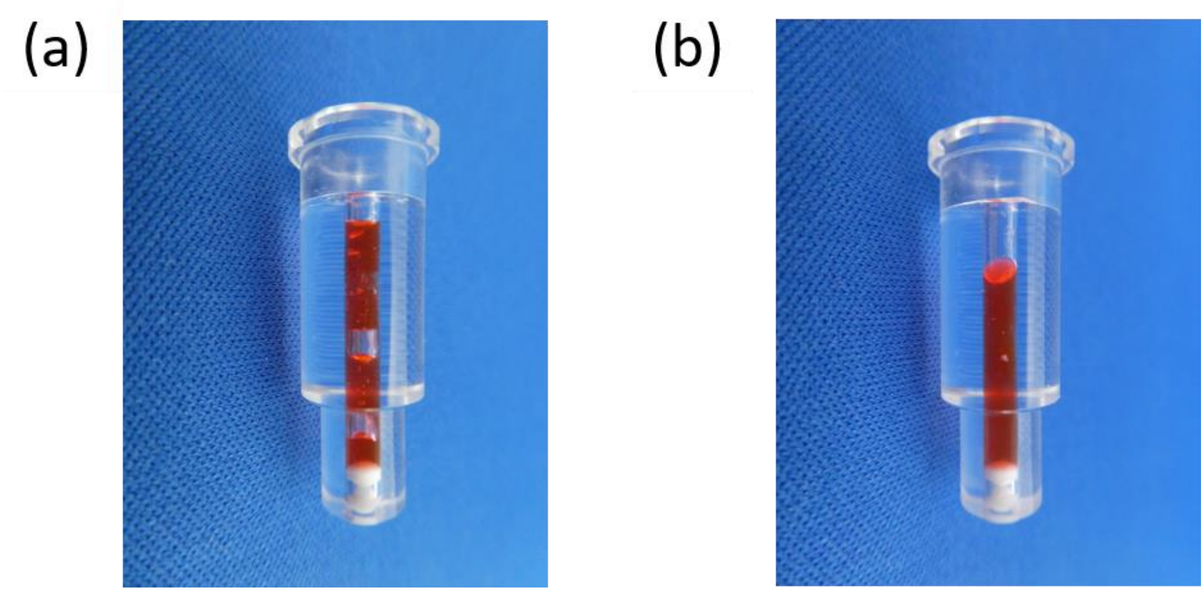

Figure S2 Sample reservoir before (a) and after (b) centrifugation. Grease colored with red ink was loaded into a 200-μL sample cartridge. The sample was centrifuged at 8000 ×*g* for 20 s. The bottom side of the cartridge was covered with a flexible film (Parafilm) to prevent samples leaking.

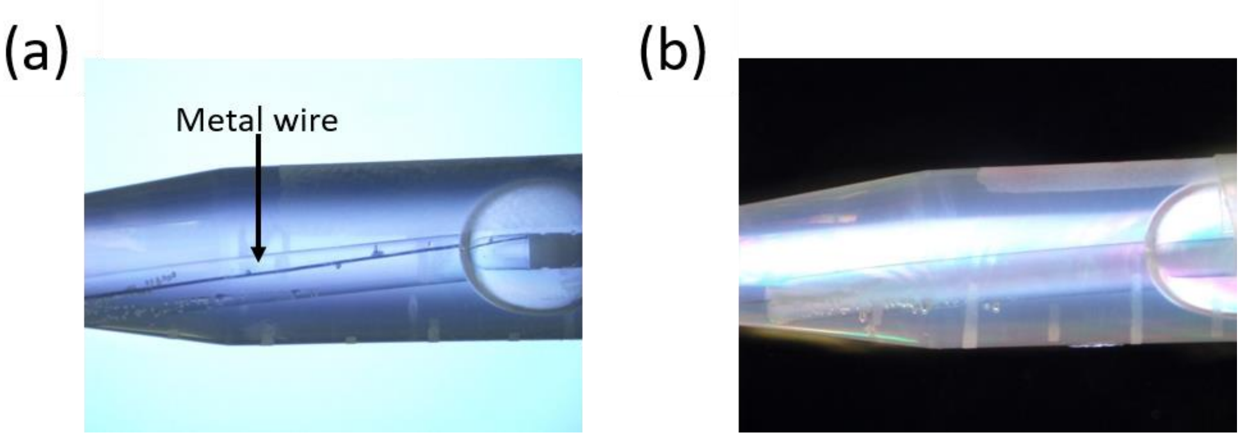

Figure S3 LCP crystallization using a metal wire. Before (a) and after (b) the appearance of crystals

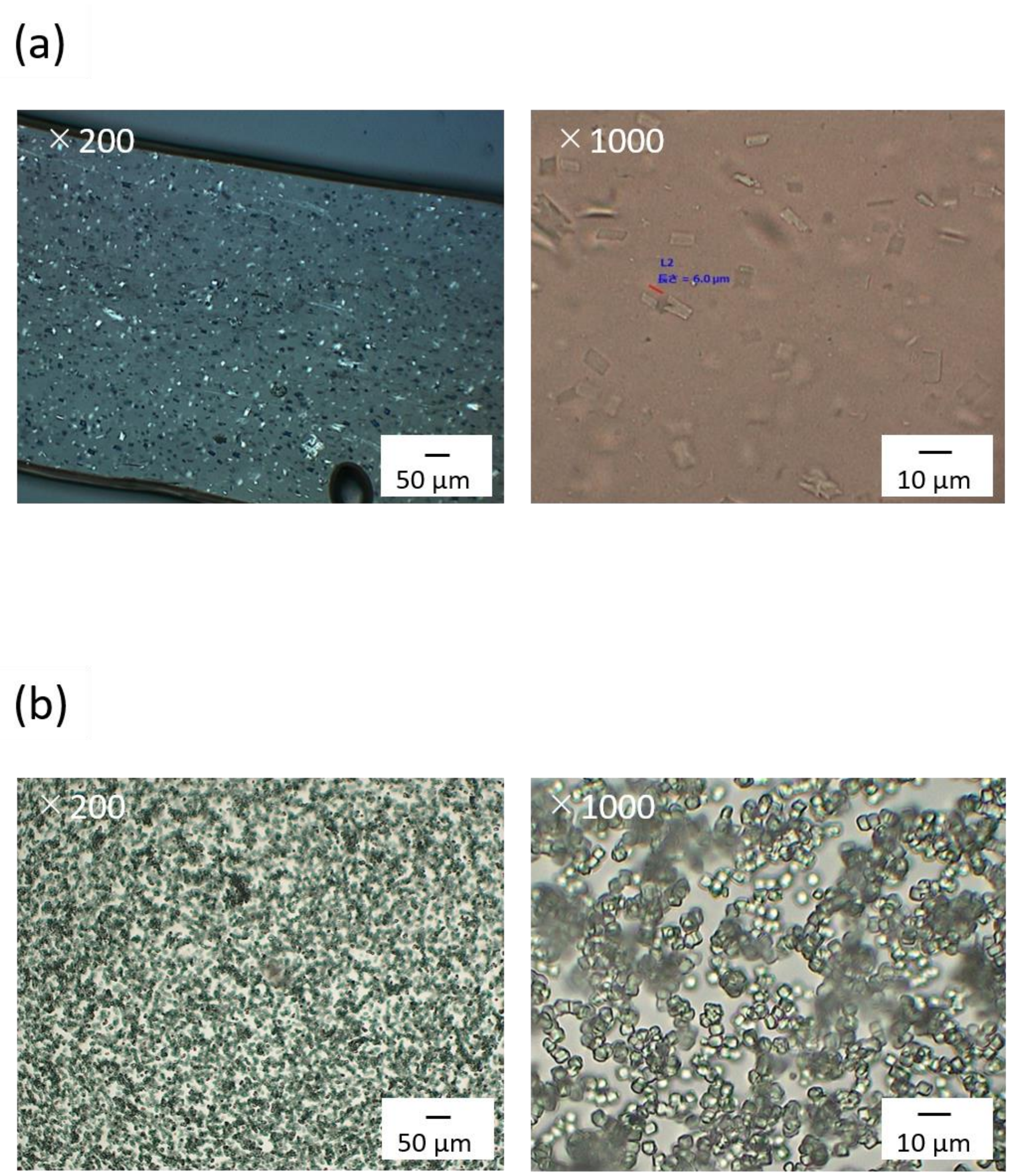

Figure S4 (a) $A_{2A}AR$ crystals in LCP used for data collection. The crystal density was estimated to be approximately one-tenth of the density of the lysozyme crystals. (b) Lysozyme crystals (~5 µm in size) used for data collection. The crystal density of 2.3 × 10^8 crystals/mL was determined by counting the crystals in a cell-counting plate.

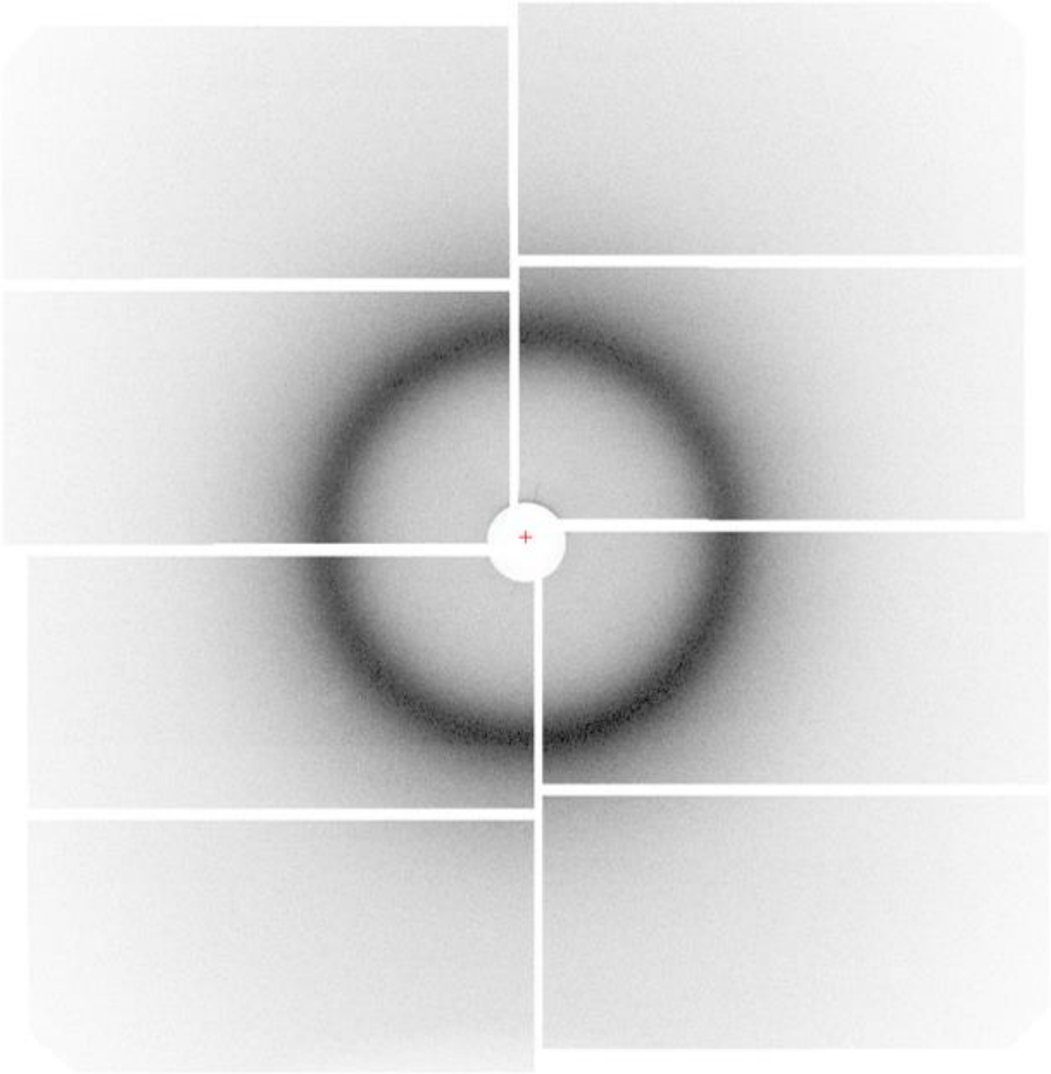

Figure S5 Representative image not counted as a hit for the $A_{2A}AR$ crystals. The image shows weak scattering patterns from the lipids.

Sample	Matrix/Sample flow rate (µl/min)	Resolution (Å)	Indexed images	PDB-ID	CXIDB	References
Human orexin-2 receptor	LCP/0.42	2.3	18,359	5WS3	74	(Suno *et al.*, 2018)
Nitric oxide reductase	Hydroxyethyl cellulose/1.25	2.0<br>2.0<br>2.1<br>2.1	34,811<br>35,309<br>108,597<br>109,519	5Y5I<br>5Y5J<br>5Y5K<br>5Y5L	63	(Tosha *et al.*, 2017)
Influenza M2 channel	LCP/0.42	1.4<br>1.45<br>1.4	34,113<br>29,173<br>63,249	5JOO<br>5UM1<br>5TTC	-	(Thomaston *et al.*, 2017)
Cytochrome c oxidase	LCP/0.48	2.3	8,211	5NDC	-	(Andersson *et al.*, 2017)
Lysozyme	Hydroxyethyl cellulose/0.43 or 0.75	1.8<br>1.45	29,593<br>40,787	5WR9<br>5WRA	50	(Sugahara *et al.*, 2017)
Lysozyme	Nuclear grade grease/0.42	2.0	19,271	5WRB	47	(Sugahara *et al.*, 2017)
Thaumatin	Hydroxyethyl cellulose/0.47	1.55	43,350	5WR8	49	(Sugahara *et al.*, 2017)
Proteinase K	Hydroxyethyl cellulose/0.47	1.50	47,503 (native), 30,930 (derivative)	5WRC	45, 48	(Sugahara *et al.*, 2017)
Photosystem II	Nuclear grade grease/5.6 or 2.8	2.35<br>2.35<br>2.50<br>2.50	64,985<br>51,482<br>54,956<br>63,711	5WS5<br>5WS6<br>5GTH<br>5GTI	-	(Suga *et al.*, 2017)
Bacteriorhodopsin	LCP/2.5	2.00<br>2.10	243,639<br>17,724<br>18,937<br>20,509	5B6V<br>5B6W<br>5H2H<br>5H2I	53	(Nango *et al.*, 2016)

			20,888<br>20,587<br>19,826<br>14,803<br>15,777<br>16,851<br>19,175<br>15,371<br>15,589<br>13,648	5H2J<br>5B6X<br>5H2K<br>5H2L<br>5H2M<br>5B6Y<br>5H2N<br>5H2O<br>5H2P<br>5B6Z		
Bacteriorhodopsin<br>(Bicelle)	Synthetic grease super lube/0.25 or 0.95	2.10	23,347	5B34	43	(Nakane, Hanashima, *et al.*, 2016)

Table S1 Successful SFX results using the HVC injector at SACLA.

References

Andersson, R., Safari, C., Dods, R., Nango, E., Tanaka, R., Yamashita, A., Nakane, T., Tono, K., Joti, Y., Bath, P., Dunevall, E., Bosman, R., Nureki, O., Iwata, S., Neutze, R. & Branden, G. (2017). *Sci Rep* **7**, 4518.

Edlund, P., Takala, H., Claesson, E., Henry, L., Dods, R., Lehtivuori, H., Panman, M., Pande, K., White, T., Nakane, T., Berntsson, O., Gustavsson, E., Bath, P., Modi, V., Roy-Chowdhury, S., Zook, J., Berntsen, P., Pandey, S., Poudyal, I., Tenboer, J., Kupitz, C., Barty, A., Fromme, P., Koralek, J. D., Tanaka, T., Spence, J., Liang, M., Hunter, M. S., Boutet, S., Nango, E., Moffat, K., Groenhof, G., Ihalainen, J., Stojkovic, E. A., Schmidt, M. & Westenhoff, S. (2016). *Sci Rep* **6**, 35279.

Nakane, T., Hanashima, S., Suzuki, M., Saiki, H., Hayashi, T., Kakinouchi, K., Sugiyama, S., Kawatake, S., Matsuoka, S., Matsumori, N., Nango, E., Kobayashi, J., Shimamura, T., Kimura, K., Mori, C., Kunishima, N., Sugahara, M., Takakyu, Y., Inoue, S., Masuda, T., Hosaka, T., Tono, K., Joti, Y., Kameshima, T., Hatsui, T., Yabashi, M., Inoue, T., Nureki, O., Iwata, S., Murata, M. & Mizohata, E. (2016). *Proc Natl Acad Sci U S A* **113**, 13039-13044.

Nango, E., Royant, A., Kubo, M., Nakane, T., Wickstrand, C., Kimura, T., Tanaka, T., Tono, K., Song, C., Tanaka, R., Arima, T., Yamashita, A., Kobayashi, J., Hosaka, T., Mizohata, E., Nogly, P., Sugahara, M., Nam, D., Nomura, T., Shimamura, T., Im, D., Fujiwara, T., Yamanaka, Y., Jeon, B., Nishizawa, T., Oda, K., Fukuda, M., Andersson, R., Bath, P., Dods, R., Davidsson, J., Matsuoka, S., Kawatake, S., Murata, M., Nureki, O., Owada, S., Kameshima, T., Hatsui, T., Joti, Y., Schertler, G., Yabashi, M., Bondar, A. N., Standfuss, J., Neutze, R. & Iwata, S. (2016). *Science* **354**, 1552-1557.

Suga, M., Akita, F., Sugahara, M., Kubo, M., Nakajima, Y., Nakane, T., Yamashita, K., Umena, Y., Nakabayashi, M., Yamane, T., Nakano, T., Suzuki, M., Masuda, T., Inoue, S., Kimura, T., Nomura, T., Yonekura, S., Yu, L. J., Sakamoto, T., Motomura, T., Chen, J. H., Kato, Y., Noguchi, T., Tono, K., Joti, Y., Kameshima, T., Hatsui, T., Nango, E., Tanaka, R., Naitow, H., Matsuura, Y., Yamashita, A., Yamamoto, M., Nureki, O., Yabashi, M., Ishikawa, T., Iwata, S. & Shen, J. R. (2017). *Nature* **543**, 131-135.

Sugahara, M., Mizohata, E., Nango, E., Suzuki, M., Tanaka, T., Masuda, T., Tanaka, R., Shimamura, T., Tanaka, Y., Suno, C., Ihara, K., Pan, D., Kakinouchi, K., Sugiyama, S., Murata, M., Inoue, T., Tono, K., Song, C., Park, J., Kameshima, T., Hatsui, T., Joti, Y., Yabashi, M. & Iwata, S. (2015). *Nat Methods* **12**, 61-63.

Sugahara, M., Nakane, T., Masuda, T., Suzuki, M., Inoue, S., Song, C., Tanaka, R., Nakatsu, T., Mizohata, E., Yumoto, F., Tono, K., Joti, Y., Kameshima, T., Hatsui, T., Yabashi, M., Nureki, O., Numata, K., Nango, E. & Iwata, S. (2017). *Sci Rep* **7**, 703.

Sugahara, M., Song, C., Suzuki, M., Masuda, T., Inoue, S., Nakane, T., Yumoto, F., Nango, E., Tanaka, R., Tono, K., Joti, Y., Kameshima, T., Hatsui, T., Yabashi, M., Nureki, O., Numata, K. & Iwata, S. (2016). *Sci Rep* **6**, 24484.

Suno, R., Kimura, K. T., Nakane, T., Yamashita, K., Wang, J., Fujiwara, T., Yamanaka, Y., Im, D., Horita, S., Tsujimoto, H., Tawaramoto, M. S., Hirokawa, T., Nango, E., Tono, K., Kameshima, T., Hatsui, T., Joti, Y., Yabashi, M., Shimamoto, K., Yamamoto, M., Rosenbaum, D. M., Iwata, S., Shimamura, T. & Kobayashi, T. (2018). *Structure* **26**, 7-19 e15.

Thomaston, J. L., Woldeyes, R. A., Nakane, T., Yamashita, A., Tanaka, T., Koiwai, K., Brewster, A. S., Barad, B. A., Chen, Y., Lemmin, T., Uervirojnangkoorn, M., Arima, T., Kobayashi, J., Masuda, T., Suzuki, M., Sugahara, M., Sauter, N. K., Tanaka, R., Nureki, O., Tono, K., Joti, Y., Nango, E., Iwata, S., Yumoto, F., Fraser, J. S. & DeGrado, W. F. (2017). *Proc Natl Acad Sci U S A* **114**, 13357-13362.

Tosha, T., Nomura, T., Nishida, T., Saeki, N., Okubayashi, K., Yamagiwa, R., Sugahara, M., Nakane, T., Yamashita, K., Hirata, K., Ueno, G., Kimura, T., Hisano, T., Muramoto, K., Sawai, H., Takeda, H., Mizohata, E., Yamashita, A., Kanematsu, Y., Takano, Y., Nango, E., Tanaka, R., Nureki, O., Shoji, O., Ikemoto, Y., Murakami, H., Owada, S., Tono, K., Yabashi, M., Yamamoto, M., Ago, H., Iwata, S., Sugimoto, H., Shiro, Y. & Kubo, M. (2017). *Nat Commun* **8**, 1585.

Movie S1 Close-up view of the sample stream from the HVC injector at beanline 3 of SACLA. Microcrystals of A_{2A}AR complexed with ZM241385 embedded in LCP were extruded from the nozzle of 75-µm ID. The sample stream was continuously supplied without curling up although the stream was chopped by the XFEL beam.
